# Lipoprotein(a), Immunity, and Inflammation in Polyvascular Atherosclerotic Disease

**DOI:** 10.3390/jcdd8020011

**Published:** 2021-01-27

**Authors:** Narek A. Tmoyan, Olga I. Afanasieva, Marat V. Ezhov, Elena A. Klesareva, Tatiana V. Balakhonova, Sergei N. Pokrovsky

**Affiliations:** 1 A.L. Myasnikov Institute of Clinical Cardiology, National Medical Research Center of Cardiology, Ministry of Health of the Russian Federation, 121552 Moscow, Russia; marat_ezhov@mail.ru (M.V.E.); tvbdoc@gmail.com (T.V.B.); 2 Institute of Experimental Cardiology, National Medical Research Center of Cardiology, Ministry of Health of the Russian Federation, 121552 Moscow, Russia; afanasieva.cardio@yandex.ru (O.I.A.); hea@mail.ru (E.A.K.); dr.pokrovsky@mail.ru (S.N.P.); 3 Department of Cardiology, Functional and Ultrasound Diagnostics, Sklifosovsky Institute of Clinical Medicine, Federal State Autonomus Educational Institution of Higher Education I.M. Sechenov First Moscow State Medical University of the Ministry of Health of the Russian Federation (Sechenov University), 119991 Moscow, Russia

**Keywords:** lipoprotein(a), hyperlipoproteinemia(a), apolipoprotein(a), autoantibodies, C-reactive protein, circulating immune complexes, atherosclerosis, carotid artery disease, atherosclerosis of lower limb arteries, coronary artery disease

## Abstract

Background and aims: lipoprotein(a) (Lp(a)) is a genetically determined risk factor for coronary artery disease and its complications, although data on the association with other vascular beds and the severity of atherosclerosis is limited. The aim of this study was to evaluate the association of atherosclerosis of various vascular beds with Lp(a), as well as its autoantibodies and generalized inflammatory markers. Material and methods: this study included 1288 adult patients with clinical and imaging examination of three vascular beds (coronary, carotid, and lower limb arteries). Patients were categorized according to the number of affected vascular beds (with at least one atherosclerotic stenosis ≥50%): 0 (*n* = 339), 1 (*n* = 470), 2 (*n* = 315), 3 (*n* = 164). We assessed blood cell count, lipid profile, C-reactive protein, circulating immune complexes, Lp(a), and its autoantibodies. Results: the number of affected vascular beds was associated with an increasing level of Lp(a) and a lower level of IgM autoantibodies to Lp(a). Hyperlipoproteinemia(a) (Lp(a) ≥ 30 mg/dL) was detected more frequently in patients with atherosclerosis. In logistic regression analysis adjusted for age, sex, hypertension, type 2 diabetes, and smoking, an elevated Lp(a) level was independently associated with stenotic atherosclerosis and lesion severity. There was a positive association of the number of affected vascular beds with C-reactive protein (*r* = 0.21, *p* < 0.01) and a negative association with circulating immune complexes (*r* = −0.29, *p* < 0.01). The neutrophil-to-lymphocyte ratio was significantly higher and the lymphocyte-to-monocyte ratio was significantly lower in patients with atherosclerosis compared to the controls (*p* < 0.01). Conclusion: Lp(a), C-reactive protein, circulating immune complexes, and neutrophil-to-lymphocyte ratio are associated with the stenotic atherosclerosis of different vascular beds. Lp(a) levels increase and IgM autoantibodies to Lp(a) decrease with the number of affected vascular beds.

## 1. Introduction

Atherosclerosis and cardiovascular diseases remain the leading cause of mortality worldwide. According to the REACH (Reduction of Atherothrombosis for Continued Health) registry, the frequency of cardiovascular death, myocardial infarction, stroke, or hospitalization for atherothrombotic complications within one year in patients with lower extremity artery disease (LEAD), coronary artery disease (CAD), or cerebrovascular disease (CVD) was 21.14%, 15.20%, and 14.53%, respectively, and increased with disease severity [1,2]. The high rate of cardiovascular events in patients with known atherosclerotic cardiovascular disease mandates more aggressive treatment strategies. Thus, the implementation of new methods of determining the probability of cardiovascular events for individuals remains a key challenge. Furthermore, it has been shown that the risk of cardiovascular events in patients with atherosclerosis of lower limb arteries is higher in the presence of CAD or CVD [3]. In addition to the high risk of cardiovascular death, myocardial infarction, and ischemic stroke, there is a higher probability of acute limb ischemia, amputation, and revascularization in patients with atherosclerosis of the lower limb arteries [4]. Lipoprotein(a) (Lp(a)) is a unique atherogenic lipoprotein particle and independent cardiovascular risk factor that is mainly associated with CAD and coronary events [5,6]. The relationship of Lp(a) with peripheral and polyvascular atherosclerotic disease has been explored insufficiently in the past, and available data are contradictory. We suggested, sought out, and found the association of Lp(a), as well as some markers of inflammation and immunity, with stenotic atherosclerosis in different vascular beds.

## 2. Material and Methods

The study included 1288 Caucasian subjects older than 18 years with clinical and instrumental examination of three vascular beds (coronary, carotid, and lower limbs arteries) hospitalized in the National Medical Research Center of Cardiology (Moscow, Russia). The local ethics committee approved the study protocol. Written informed consent forms were obtained from all patients prior to enrollment. The study was conducted in accordance with good clinical practice and the principles of the Declaration of Helsinki. Exclusion criteria were acute coronary syndrome; infectious and inflammatory diseases during the last 3 months; chronic heart failure, III–IV functional class; rheumatoid disease; chronic kidney disease, stage IV or V; severe thyroid dysfunction (thyroid-stimulating hormone >2 times the lower or upper limit of normal); acute hepatitis, liver cirrhosis; and therapy affecting the level of Lp(a) (apheresis, niacin, PCSK9 inhibitors, glucocorticoids, or hormones).

Stenotic atherosclerosis was defined as ≥50% stenosis in at least one coronary, carotid, or lower limb artery on an ultrasound or angiographic study. The patients were divided into four groups, depending on the number of the affected vascular beds: group 0, without stenotic atherosclerosis, *n* = 339; group I, stenotic atherosclerosis of one vascular bed, *n* = 470; group II, stenotic atherosclerosis of two vascular beds, *n* = 315; and group III, stenotic atherosclerosis of three vascular beds, *n* = 164. CAD, CVD, and LEAD were defined as the stenotic atherosclerosis of coronary, carotid, or lower extremity arteries, respectively.

The concentration of total cholesterol (TC), triglycerides (TGs), and high-density lipoprotein cholesterol (HDL-C) was determined by enzymatic colorimetric method on an Architect C 8000 analyzer (Abbott, United States). Low-density lipoprotein cholesterol (LDL-C) levels were calculated according to the Friedewald formula and its modification [7]: LDL-C_corrected_ = TC − HDL-C − (TG/2.2) − (0.3 × Lp(a)/38.7), where LDL-C_corrected_ is the level of LDL-C adjusted for Lp(a) cholesterol. The serum concentration of Lp(a) was measured with an enzyme-linked immunosorbent assay (ELISA), using polyclonal, monospecific sheep antibodies against human Lp(a) [8]. The method was validated with two kits: TintElize Lp(a) (Biopool AB, Umea, Sweden) and Immunozym Lp(a) (Progen Biotechnik GmbH, Heidelberg, Germany). Control samples approved by the International Federation of Clinical Chemistry (Technoclone, Vienna, Austria), and were used to standardize the ELISA. The levels of specific autoantibodies against apoB100-containing lipoproteins were determined by ELISA in the serum of 512 randomly selected representative patients, with plates coated by purified Lp(a) or LDL, according to what has been previously described [9]. Goat antibodies to human IgG or IgM (specific to the γ-chain and μ-chain, respectively) conjugated to horseradish peroxidase were used as developing antibodies. The level of C-reactive protein (CRP) in blood serum was determined using a highly sensitive ELISA kit CRP-EIA-BEST (Vector-best, Novosibirsk, Russia). The specificity of the test was ensured using monoclonal antibodies against CRP. The sensitivity of the test was 0.05 IU/L, with a measurement range of 0–10 IU/L. The inter-assay coefficient of variation did not exceed 8%. The linearity of the method is 90–110% in the range of concentration of calibration samples from 1–10 IU/L. The conversion factor of IU/L to mg/L is 1.02. Calibration and control samples were certified according to the World Health Organization (WHO) International Standard for Human C-Reactive Protein, the first international standard of the National Institute for Biological Standards and Control (NIBSC) 85/506. Circulating immune complexes (CICs) were measured by an immunoturbidimetric assay with a CIC-Xema reagent kit (Xema-Medica Co. Ltd., Moscow, Russia) for the determination of circulating immune complexes in human serum or plasma. The test is based on selective polyethylene glycol (PEG) 6000 precipitation of the CIC method in microwells. The final concentration of PEG in the samples was 4%. CIC level was determined by the comparison of optical density values in wells with and without PEG at 450 nm. The coefficient of variation in the CIC-Xema kit did not exceed 8.0%; the recommended upper limit for healthy individuals is 120 lab units.

Neutrophil-to-lymphocyte, platelet-to-lymphocyte, and lymphocyte-to-monocyte ratios were calculated as the ratio of the absolute number of the corresponding cell parameters [10].

Statistical analysis of the results was conducted with MedCalc 15.8 software (MedCalc Software Ltd., Ostend, Belgium). Descriptive statistics for continuous variables are presented as the median with interquartile or 95% confidence interval (CI). Analytical statistics were performed using Mann–Whitney and Kruskal–Wallis tests. Fisher’s exact test was used for comparison of the frequency indices between the groups. To assess the associations between evaluated parameters and the presence of stenotic atherosclerosis, odds ratios (ORs) were calculated with 95% CI. To analyze the relationship between independent variables and atherosclerosis, Spearman’s correlation was applied. The threshold values of Lp(a) and the age for the prediction of stenotic atherosclerosis were determined with receiving operating characteristics curves (ROC analysis). Multivariate analysis was done with logistic regression; the model included risk factors that demonstrated an association with stenotic atherosclerosis in univariate analysis. Creating the model, the absence of internal correlations between estimated parameters was taken into account. The differences were considered statistically significant at *p* < 0.05.

## 3. Results

### 3.1. General Characteristics of Patients

The general characteristics of the patients are presented in Table 1. Patients with stenotic atherosclerosis (groups I, II, and III) were older than individuals without stenotic atherosclerosis (group 0). There were more men, as well as higher frequency of hypertension and smoking. The frequency of type 2 diabetes was higher in the groups with stenotic atherosclerosis of two or three vascular beds than in the group without stenotic atherosclerosis. The levels of TC, LDL-C, and LDL-C_corrected_ were lower in groups with stenotic atherosclerosis. The level of TG was lower in groups II and III when compared with group 0. Statins were taken more often in groups I–III compared to group 0 (Table 1).

### 3.2. The Association of Lp(a) and Other Markers with Atherosclerosis

**Figure 1:**
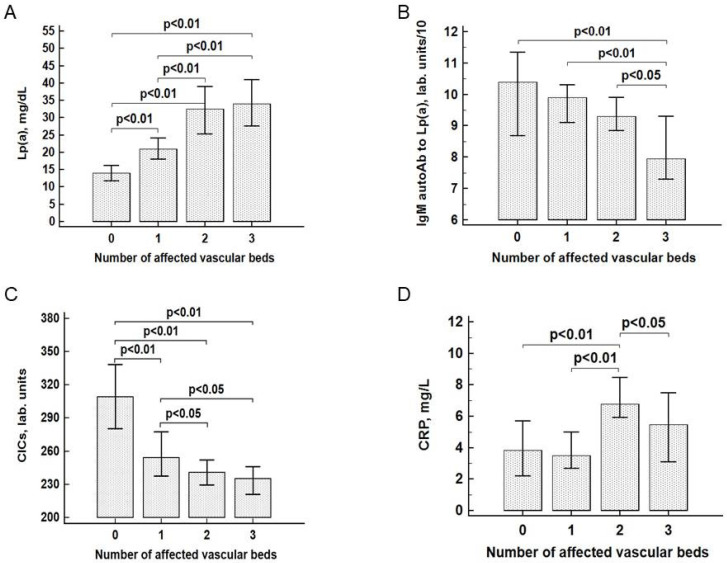
(**A**) The levels of lipoprotein(a). (**B**) IgM autoantibodies to lipoprotein(a) (the levels of specific autoantibodies against Lp(a) and LDL were determined in the serum of 512 randomly selected representative patients: group 0, *n* = 72; group I, *n* = 153; group II, *n* = 183; group III, *n* = 104). (**C**) Circulating immune complexes. (**D**) C-reactive protein, depending on the number of affected vascular beds. Data are presented as the median and 95% confidence interval. Lp(a): lipoprotein(a), CICs: circulating immune complexes, CRP: C-reactive protein, autoAb: autoantibody.

The concentration of Lp(a) was positively correlated with the number of vascular beds affected by stenotic atherosclerosis (Figure 1A). The level of Lp(a) in patients with stenotic atherosclerosis of two or three vascular beds was significantly higher than in patients with isolated involvement of one vascular bed (coronary, carotid, or lower limb arteries) or non-stenotic atherosclerosis. The concentration of Lp(a) was not associated with the level of blood lipids (TC, TG, HDL-C, LDL-C), sex, age, presence of hypertension, or type 2 diabetes. A significant correlation was found between Lp(a) and the number of affected vascular beds (*r* = 0.25, *p* < 0.01) and CRP (*r* = 0.13, *p* = 0.01). The level of IgM autoantibodies specific for Lp(a) decreased with the number of affected vascular beds (Figure 1B). The minimal serum level of IgM autoantibodies to Lp(a) was observed in patients with stenotic lesions of all three vascular beds, while the differences were significant compared to persons without stenotic atherosclerosis and patients with stenotic atherosclerosis of one or two vascular beds (*p* < 0.05 in all cases). The level of IgM autoantibodies to Lp(a) was negatively associated with the number of affected vascular beds (*r* = −0.11; *p* = 0.01). There were no differences in the level of IgG autoantibodies against Lp(a) and LDL, or of IgM autoantibodies against LDL between the groups.

Correlation analysis showed a positive association between the number of stenotic atherosclerosis vascular beds with CRP (*r* = 0.21; *p* < 0.01) and a negative association with CICs (*r* = −0.29; *p* < 0.01). The level of CIC was significantly higher in controls compared to patients with stenotic atherosclerosis, and lower with the number of affected vascular beds (Table 1, Figure 1C). The level of CRP was higher in patients with atherosclerosis of two or three vascular beds compared to patients without atherosclerosis or atherosclerosis of one vascular bed (Figure 1D).

The neutrophil-to-lymphocyte ratio was significantly higher and lymphocyte-to-monocyte ratio was significantly lower in patients with atherosclerosis compared to the controls (Table 1).

According to the ROC analysis, patients over 64 years of age had a sensitivity of 61% and specificity of 77% (area under the curve = 0.76; 95% CI = 0.73–0.79, *p* < 0.01), and the concentration of Lp(a) ≥30 mg/dL had a sensitivity of 53% and specificity of 75% (area under the curve = 0.64; 95% CI = 0.61–0.67, *p* < 0.01) to detect atherosclerotic lesions.

**Figure 2:**
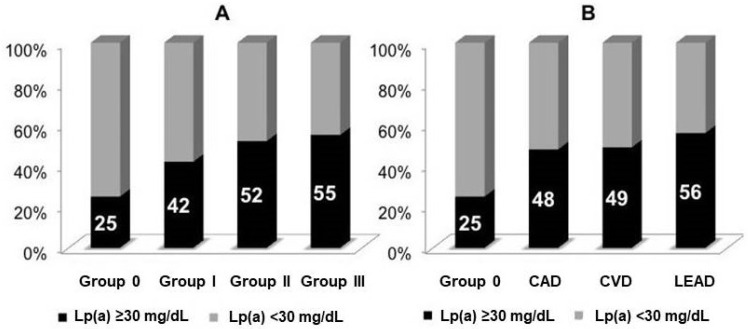
The distribution of elevated level of lipoprotein(a) (**A**) depending on the number of affected vascular beds, (**B**) in patients with different cardiovascular diseases. CAD: coronary artery disease, CVD: cerebrovascular disease, LEAD: lower extremity artery disease.

The level of Lp(a) ≥30 mg/dL (hyperlipoproteinemia(a)) was observed in 42% of patients. Hyperlipoproteinemia(a) was detected more frequently in groups with stenotic atherosclerosis compared to group 0 (Figure 2A). According to logistic regression analysis, an elevated Lp(a) level shows an independent association with stenotic atherosclerosis and lesion severity (Table 2); levels of CRP and IgM autoantibodies against Lp(a) were not associated with atherosclerosis, whereas there was an inverse association between the CICs and stenotic atherosclerosis.

According to logistic regression analysis, the elevation of the Lp(a) concentration by 1 mg/dL led to increasing the probability of one-bed stenotic atherosclerosis by 1% (OR = 1.01 (95% CI = 1.00–1.02), *p* < 0.01), stenotic atherosclerosis of two beds by 2% (OR = 1.02 (95% CI = 1.02–1.03), *p* < 0.01), and stenotic atherosclerosis of three beds by 3% (OR = 1.03 (95% CI = 1.02–1.04), *p* < 0.01). The CIC level, on the contrary, was inversely associated with atherosclerosis: the OR of stenotic atherosclerosis of one bed was 0.99 (95% CI = 0.98–0.99; *p* < 0.01), of two beds was 0.98 (95% CI = 0.98–0.99; *p* < 0.01), and of three beds was 0.97 (95% CI = 0.95–0.99; *p* < 0.01).

**Figure 3:**
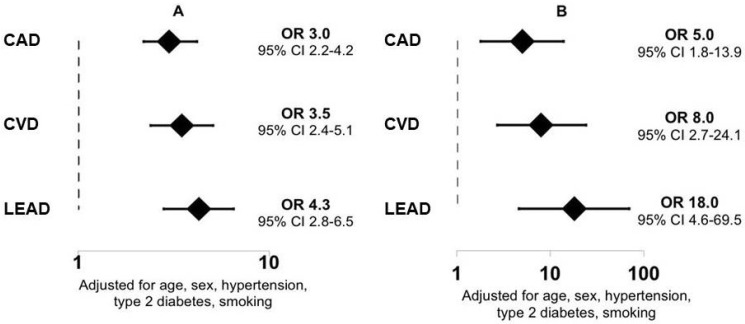
Odds ratios of cardiovascular diseases (**A**) in the case of hyperlipoproteinemia(a), and (**B**) in the case of hyperlipoproteinemia(a) and the level of IgM autoantibodies against lipoprotein(a) being below the median. Data are presented as odds ratio (ORs) and 95% confidence intervals. CAD: coronary artery disease, CVD: cerebrovascular disease, LEAD: lower extremity artery disease.

In this cohort, CAD was diagnosed in 801 patients, CVD in 465 patients, and LEAD in 326 patients. Hyperlipoproteinemia(a) was detected more frequently in patients with CAD, CVD, and LEAD compared to patients without stenotic atherosclerosis (Figure 2B). According to logistic regression analysis, the level of Lp(a) ≥30 mg/dL remained an independent risk factor for CAD, CVD, and LEAD (Figure 3A).

Myocardial infarction in the past was observed in 266 (49%) of 538 individuals with a Lp(a) level ≥30 mg/dL and in 277 (37%) of 750 patients with a Lp(a) level <30 mg/dL (*p* < 0.01). Myocardial infarction in the past was associated with hyperlipoproteinemia(a) with OR = 1.7 (1.3–2.1). Lp(a) ≥30 mg/dL was detected in 44% of patients with ischemic stroke, but only in 26% of the subjects in the control group (*p* < 0.01), and was associated with those who had suffered a stroke, with OR = 2.3 (1.5–3.5; *p* < 0.01).

### 3.3. Lp(a), IgM Autoantibodies, and Cardiovascular Diseases

The level of IgM autoantibodies against Lp(a) was significantly higher in patients without severe atherosclerosis compared to patients with CAD, CVD, and LEAD, at 104 (80; 128) vs 94 (73; 115), 90 (71; 116), and 87 (70; 114) laboratory units (lab. units), respectively (*p* < 0.05 for all). Hyperlipoproteinemia(a) was associated with ORs of 3.0, 3.5, and 4.3 for CAD, CVD, and LEAD, respectively. The combination of hyperlipoproteinemia(a) and a low level of IgM autoantibodies against Lp(a) (less than the median of 94 lab. units) increased the ORs of CAD, CVD, and LEAD significantly (Figure 3B).

## 4. Discussion

For the first time in a cross-sectional study of 1288 patients, we have shown the gradual increase of Lp(a) concentration with the number of affected vascular beds. Higher levels of Lp(a) are associated with more severe atherosclerosis involving multiple vascular beds. The EUCLID (Examining Use of Ticagrelor in Peripheral Artery Disease) study has demonstrated the increasing risk of cardiovascular complications in patients with LEAD, with each additional lesion of other vascular beds [11]. Those and our results indicate the necessity of the examination of various vascular beds, with the aim of early treatment and reduction of the risk of cardiovascular events.

This cohort included a large number of patients with elevated Lp(a). In a study conducted in the United States, with the participation of over 530,000 subjects, the level of Lp(a) >30 mg/dL was found in every third person [12]. In our study, the high frequency of hyperlipoproteinemia(a) was due to the inclusion of patients from one center, among whom there were more patients with cardiovascular diseases than in the general population. We have shown elevated Lp(a) levels in 48%, 49%, and 56% of patients with stenotic atherosclerosis of the coronary, carotid, and lower limb arteries, respectively, compared to 25% of individuals without significant atherosclerosis, who were examined in our center. Many studies have shown that an increased concentration of Lp(a) is an independent risk factor for coronary atherosclerosis and myocardial infarction [5,6]. Studies exploring the relationship of Lp(a) with atherosclerosis of the lower limb and carotid arteries are scarce. Some of them have shown the association between elevated concentrations of Lp(a) and atherosclerosis of lower limb arteries, which is included a small number of observations [13,14,15,16]. A genome-wide association study (31,307 patients with peripheral artery disease and 211,753 controls) identified 19 peripheral artery disease loci; 11 of the 19 loci were associated with disease in three vascular beds (coronary, carotid, and lower extremity), including *LDLR*, *LPL*, and *LPA.* The strongest association with peripheral artery disease was found in gene *LPA*, with OR = 1.26 [17]. Some studies have shown an association between Lp(a) and carotid atherosclerosis, but in other studies there no association was found [18,19,20,21,22,23].

It is well-known that inflammation, as well as innate and adaptive immunity play an important role in the development and progression of atherosclerosis [24,25]. Our study evaluated the contribution of Lp(a) as a possible antigen in the development of stenotic atherosclerosis, by assessing the level of circulating autoantibodies against Lp(a). For the first time, we found the relationship between higher Lp(a) levels with lower IgM autoantibodies against Lp(a) levels with stenotic atherosclerosis of multiple vascular beds. So-called “natural” IgM antibodies are produced by B1 lymphocytes to protect the human from various pathogens. These antibodies are opsonins—they recognize specific epitopes on oxidized phospholipids, which are dying cells proceeding through apoptosis, and output the formed immune complexes from the bloodstream, thus “cleansing” them from the body [26,27,28]. It has been shown that this kind of IgM antibodies present in cord blood, and their level gradually reduces with age, decreasing the ability of B1 lymphocytes to produce IgM antibodies, leading to increased cardiovascular risk [25].

The presence of a high level of specific IgM autoantibodies against Lp(a) in patients without significant atherosclerosis, compared to patients with stenotic lesions of carotid and lower limbs arteries, is consistent with an Australian study that demonstrated that IgM autoantibodies to oxidize LDL have protective effect [29]. A 15-year prospective study found that a higher level of IgM autoantibodies against LDL, modified by malondialdehyde (MDA-LDL), was associated with a low risk of acute coronary syndrome (relative risk (RR) = 0.79, 95% CI = 0.66–0.95, *p* = 0.01) and newly diagnosed cardiovascular disease (RR = 0.69, 95% CI = 0.50–0.94, *p* = 0.02) [30]. According to another prospective study, IgG autoantibodies against MDA-LDL were independently associated with the development of cardiovascular events (hazard ratio = 1.76, 95% CI = 1.16–2.72, *p* < 0.01 for the fourth quartile versus the first quartile) at a median follow-up of 10.5 years [31]. However, in another study with 2471 patients, IgM and IgG autoantibodies against MDA-LDL were not associated with CAD [32].

Available data suggest that elevated levels of IgG autoantibodies are directly associated with cardiovascular diseases, while an elevated level of IgM autoantibodies has a protective effect. Lp(a) may be one of the autoantigens, the removal of which from the bloodstream by the IgM autoantibodies may also exert a cardio-protective effect. Searching for the inflammatory mechanisms of action of Lp(a) is a promising scientific direction, and may contribute to the inflammatory process [33,34].

Blood cells, especially subpopulations of leukocytes, can be used as indicators of systemic inflammation. Neutrophils, lymphocytes, and monocytes play an important role in inflammatory response. It has been shown that both high numbers of monocytes and low number of lymphocytes are associated with the severity of coronary atherosclerosis [35], which is corroborated by our study results. We found a greater number of monocytes and neutrophils, but not lymphocytes in subjects with stenotic atherosclerosis, than in the control group. The neutrophil-to-lymphocyte ratio increased, and the lymphocyte-to-monocyte ratio decreased with the number of affected vascular beds. In a prospective study conducted in Italy in 500 patients with stable CAD neutrophil-to-lymphocyte ratios, these rations were independent predictors of such cardiovascular events as death, myocardial infarction, and repeat revascularization during five years of follow-up [36]. In that and our study, there was no difference in the platelet-to-lymphocyte ratio. In 2842 healthy Korean individuals, the neutrophil-to-lymphocyte ratio was an independent predictor of intracranial stenotic atherosclerosis [37]. Thus, neutrophil-to-lymphocyte and lymphocyte-to-monocyte ratios, as markers of systemic inflammation, are associated with atherosclerosis of the coronary, carotid, and lower limbs arteries.

We revealed a positive association between atherosclerosis and CRP, and a negative association between atherosclerosis and CICs. Elevated concentration of CRP as a biomarker of immune response and inflammation has been associated with an increased risk of cardiovascular events [38]. The role of humoral and cellular immunity in the development of atherosclerosis and its complications, as well as the search for new diagnostic tools and therapeutic approaches aimed at combating inflammation, confirm our conclusions for future directions. [39,40].

Our study has limitations. The ELISA method for Lp(a) determination was sensitive to apolipoprotein(a) isoforms, resulting in a slight increase in Lp(a) concentration in samples with high molecular weight apo(a) isoforms. The absolute bias (median (25%; 75%)) was ~1.5 (−0.4 to 5.7) mg/dL. The high variability in the Lp(a) measurement, regardless of apo(a) isoforms, and the nonsignificant bias in the absolute Lp(a) concentration in our ELISA method allows us to assume that sensitivity of ELISA to apo(a) isoforms did not affect the results of our study. In addition, there were not significant differences in the Lp(a)-associated relative risk of CAD in studies using methods sensitive and insensitive to the size of apo(a) isoforms, according to meta-analysis [5].

Also, we did not evaluate the concentration of circulating oxidized LDL, as well as autoantibodies to Cu^2+^-oxidized LDL or MDA-modified LDL.

## 5. Conclusions

Lp(a), C-reactive protein, circulating immune complexes, and the neutrophil-to-lymphocyte ratio are associated with stenotic atherosclerosis of different vascular beds. Lp(a) levels increased and IgM autoantibodies against Lp(a) decreased with the number of affected vascular beds.

## Figures and Tables

**Table 1:** General characteristics of examined patients.

Parameters	Group 0 *n* = 339	Group I *n* = 470	Group II *n* = 315	Group III *n* = 164
Age, years	57 (48; 64)	59 (51; 65) *	66 (59; 74) **	69 (64; 76) **
Male sex	151 (44%)	370 (78%) **	236 (74%) **	138 (84%) **
Hypertension	195 (57%)	339 (72%) **	273 (86%) **	148 (90%) **
Smoking	94 (27%)	251 (53%) **	164 (52%) **	101 (61%) **
Type 2 diabetes	49 (14%)	74 (15%)	99 (31%) **	61 (37%) **
Family history	80 (24%)	126 (27%)	80 (25%)	45 (27%)
TC, mmol/L	6.0 (5.1; 6.9)	5.6 (4.4; 6.8) **	4.6 (3.7; 6.0) **	4.4 (3.9; 5.2) **
TG, mmol/L	1.8 (1.3; 2.4)	1.7 (1.2; 2.4)	1.5 (1.1; 2.1) **	1.4 (1.1; 2.0) **
HDL-C, mmol/L	1.2 (1.1; 1.5)	1.1 (1.0; 1.3) **	1.1 (0.9; 1.3) **	1.1 (1.0; 1.3) **
LDL-C, mmol/L	3.9 (3.0; 4.7)	3.4 (2.4; 4.5) **	2.7 (2.0; 3.7) **	2.5 (2.1; 3.1) **
LDL-C_corrected_, mmol/L	3.7 (2.8; 4.4)	3.1 (2.1; 4.2) **	2.2 (1.6; 3.4) **	2.2 (1.7; 2.8) **
Statin therapy	79 (23%)	381 (81%) **	286 (91%) **	154 (94%) **
CRP, mg/L	3.9 (1.2; 8.9)	3.5 (1.7; 7.7)	6.8 (4.0; 15.5) **	5.5 (2.3; 9.3)
CICs, lab units	309 (250; 372)	254 (216; 310) **	241 (204; 279) **	235 (192; 264) **
Neutrophil-to-lymphocyte ratio	1.4 (1.1; 2.1)	1.7 (1.4; 2.3) **	1.9 (1.5; 2.4) **	1.8 (1.5; 2.6) **
Platelet-to-lymphocyte ratio	99.0 (83.6; 123.5)	102.3 (77.7; 124.6)	102.4 (78.9; 125.4)	102.9 (80.6; 123.3)
Lymphocyte-to-monocyte ratio	4.3 (3.7; 5.7)	3.9 (3.0; 5.0) *	3.6 (2.9; 4.6) **	3.8 (3.0; 4.9) **
Absolute monocytes, ×10^9^/L	0.49 (0.43; 0.65)	0.55 (0.46; 0.68) *	0.60 (0.49; 0.71) **	0.56 (0.46; 0.70)*
Absolute neutrophils, ×10^9^/L	3.2 (2.5; 4.2)	3.8 (3.1; 4.8) **	4.3 (3.4; 5.2) **	4.1 (3.2; 5.0) **
Absolute lymphocytes, ×10^9^/L	2.2 (1.8; 2.7)	2.2 (1.7; 2.6)	2.2 (1.8; 2.7)	2.2 (1.7; 2.7)
WBC, ×10^9^/L	6.4 (5.2; 7.6)	6.7 (5.8; 8.0) *	7.3 (6.2; 8.8) **	6.9 (6.0; 8.4) **

Note. Data are presented as absolute number of patients (%) or median (25th; 75th percentile). TC: total cholesterol; TG: triglyceride; HDL-C: high-density lipoprotein cholesterol; LDL-C: low-density lipoprotein cholesterol, LDL-C_corrected_: low-density lipoprotein cholesterol, corrected for lipoprotein(a) cholesterol; CRP: C-reactive protein; WBC: white blood cells CICs: circulating immune complexes. * *p* < 0.05, ** *p* < 0.01 compared to group 0.

**Table 2:** The odds ratios of stenotic atherosclerosis, depending on risk factors.

Parameters	Group 0 *n* = 339	Group I *n* = 470	Group II *n* = 315	Group III *n* = 164
Age ≥ 64 years	1	1.6 (1.2–2.4) *	4.1 (2.7–6.2) *	8.7 (4.9–15.6) *
Male sex	1	4.6 (3.2–6.6) *	4.8 (3.1–7.6) *	10.2 (5.0–20.8) *
Hypertension	1	2.2 (1.5–3.1) *	4.1 (2.6–6.5) *	6.8 (3.3–14.0) *
Type 2 diabetes	1	1.1 (0.7–1.8)	2.8 (1.7–4.5) *	3.3 (1.8–6.2) *
Smoking	1	1.9 (1.4–2.7) *	2.3 (1.5–3.5) *	3.1 (1.7–5.6) *
Lp(a) ≥ 30 mg/dL	1	2.3 (1.7–3.3) *	3.5 (2.3–5.2) *	6.1 (3.4–10.9) *

* *p* < 0.01 compared to group 0. Results of logistic regression analysis.

## Data Availability

The data presented in this study are available on request from the corresponding author.
